# Formal comment to Toyota et al.: Short versus prolonged dual antiplatelet therapy (DAPT) duration after coronary stent implantation: A comparison between the DAPT study and 9 other trials evaluating DAPT duration

**DOI:** 10.1371/journal.pone.0184513

**Published:** 2017-09-20

**Authors:** Sammy Elmariah

**Affiliations:** 1 Cardiology Division, Department of Medicine, Massachusetts General Hospital, Harvard Medical School, Boston, Massachusetts, United States of America; 2 Baim Institute for Clinical Research, Boston, Massachusetts, United States of America; UMCU, NETHERLANDS

I read with great interest the article by Toyota and colleagues published in PLOS ONE entitled, “Short versus prolonged dual antiplatelet therapy (DAPT) duration after coronary stent implantation: A comparison between the DAPT study and 9 other trials evaluating DAPT duration”.[1] The authors compare results of the Dual Antiplatelet Therapy Study (DAPT Study) to pooled results of 9 other randomized trials evaluating duration of DAPT. The analysis concludes that the beneficial effects of prolonged DAPT in reducing rates of myocardial infarction (MI) and stent thrombosis (ST) seen within the DAPT Study were greater than in the other pooled trials, yet the magnitude of excess risk associated with prolonged DAPT was consistent across studies. In part, the authors suggest that this discrepancy may be due to higher rates of MI and ST within the control arm of the DAPT Study compared to the control arms of the other studies.

While the desire to further understand the risks and benefits of prolonged DAPT holds merit, several points need to be considered when interpreting the presented results. First and foremost, the DAPT Study is a large, international, multicenter, randomized, double-blinded, placebo-controlled trial powered specifically to detect the impact of prolonged DAPT on the co-primary efficacy endpoints of definite or probably stent thrombosis and of major cardiovascular and cerebrovascular events.[2] The DAPT study therefore represents the gold standard of clinical experimental evidence. The comparator, on the other hand, is a pooled analysis of 9 heterogeneous randomized trials, none of which was statistically powered to study MI or ST rates. Each of the studies, with the exception of ISAR-SAFE,[3] were open label trials without placebo control, raising the possibility of inherent bias. Moreover, only 2 of the 9 studies (DES LATE and ARCTIC-Interruption) evaluated the clinical impact of prolonged DAPT (18 to 36 months) relative to the standard 12 months of therapy,[4, 5] as did the DAPT Study.[2] The other 7 trials, in contrast, focused on the safety of early DAPT discontinuation (3 or 6 months) after percutaneous coronary intervention (PCI) compared to 12 months of therapy. The authors’ analysis assumes that the magnitude of risk reduction remains constant from 3 to 36 months after PCI, an assumption that is not supported by the literature. Within a recent analysis from the DAPT study, rates of MI and ST and the relative risk reduction seen with prolonged DAPT 15–30 months after PCI were approximately half of those 12–15 months after PCI.[6]

The authors posit that the robust reduction in ST and MI risks observed with prolonged therapy within in the DAPT Study is due to higher baseline rates of MI and ST, therefore insinuating that the DAPT Study cohort is a higher risk cohort that may benefit of more prolonged antiplatelet therapy. This point is further supported by the fact that a greater proportion of subjects within the DAPT study had undergone prior coronary revascularization prior to study enrollment, although subgroup analyses of the DAPT Study actually demonstrate numerically greater risk reduction with prolonged DAPT in those without prior revascularization.

Regardless of the aforementioned points, I agree with the authors that the DAPT duration should be personalized to target prolonged therapy in those at greatest risk for ischemic events and abbreviated therapy in those at high risk of bleeding. Short-duration DAPT appears to be acceptably safe after contemporary DES use. In patients with hemorrhagic complications, at high risk of bleeding, or needing chronic anticoagulation, a short-duration DAPT is prudent, assuming patients are not concurrently at high risk for ischemic complications. In the absence of bleeding events or risk, the default duration of DAPT should be 12 months. Beyond 12 months of follow-up, the balance of hemorrhagic and ischemic risks should be reconsidered. Based on the proven efficacy and safety of prolonged DAPT,[2, 7] DAPT should preferably continue to 30 months to minimize risk of ST and non-stent-related MI, especially in those with recurrent ischemic events or at high risk of subsequent events. The recently developed DAPT score may provide further guidance for patients that do not fall within the extremes of risk.[8] Using five clinical factors and three index procedural characteristics (score range, -2 to 10), the score estimates the balance between ischemic and bleeding risks in patients who have remained free of ischemic or bleeding events 1 year after DES. Patients with scores < 2 possess a risk of bleeding that outweighs ischemic risk; whereas, with scores ≥ 2, ischemic risk outweighs bleeding risk. The DAPT score therefore answers the call by Toyota and colleagues by providing a means to personalize DAPT to fit individual patients.
